# Case report: transient small bowel intussusception presenting as right lower quadrant pain in a 6-year-old male

**DOI:** 10.1186/2036-7902-6-7

**Published:** 2014-05-22

**Authors:** Mathew J Nelson, Tara Paterson, Christopher Raio

**Affiliations:** 1 Department of Emergency Medicine, North Shore University Hospital, 300 Community Drive, Manhasset, NY 11030, USA

## Abstract

In children presenting to the emergency room with right lower quadrant pain, ultrasound is the preferred initial modality. In our patient, a 6-year-old male with a sudden onset of severe right lower quadrant pain, the differential is broad, including appendicitis and intussusception. In order to narrow our differential and secure the diagnosis, our first modality was ultrasonography. With the increased use of point-of-care ultrasound in the emergency department, the diagnosis of appendicitis and ileo-colic intussusception has been made more frequently. In addition, other entities such as transient small bowel intussusception may be identified. As in our case, obstruction secondary to intussusception must be ruled out with observation, serial abdominal exams, clinical improvement, or further imaging.

## Background

Abdominal pain is a very common presenting complaint to the emergency department. The most common surgical cause of right lower quadrant pain is appendicitis; however, there are many other causes that must be considered in the differential diagnosis. There are inflammatory and infectious causes involving the ileocecal region, omentum, epiploic appendages, mesentery, and other miscellaneous conditions [1]. Intussusception, omental infarction, mesenteric adenitis, epiploic appendagitis, lymphoma, and adenocarcinoma may all present with right lower quadrant pain; therefore, advanced imaging modalities such as ultrasonography and computerized tomography can aid in definitive diagnosis.

## Case presentation

A 6-year-old male presented to the emergency department (ED) with sudden onset, 10/10, constant, sharp, non-radiating right lower quadrant abdominal pain of 4 h in duration. According to his mother, he had no nausea, vomiting, fever, or chills. His last normal bowel movement was 1 day prior, and he denied any urinary complaints. There was no history of rash, sick contacts, or recent travel.

### Physical examination

The patient was a well-developed, young male in moderate distress. Vital signs included a blood pressure of 107/66 mm Hg, pulse of 110 beats per minute, respiratory rate of 24 breaths per minute, temperature of 96.9°F, and oxygen saturation of 95% on room air. His skin was dry and warm to palpation. The oropharynx was erythematous, with no tonsillar enlargement or exudates. The patient was tachycardic, but no murmurs, rubs, or gallops were auscultated. His abdomen was soft and non-distended, tender to palpation in the right lower quadrant with rebound and guarding; normal active bowel sounds were present. There was no costovertebral angle tenderness present. Rectal exam was normal with hemoccult-negative brown stool. The genitourinary exam was also unremarkable. The remainder of the physical exam was non-contributory.

### Laboratory results

The only lab findings of note were hypokalemia to 3.3 mmol/L and bicarbonate level of 17 mmol/L. The white blood cell count was normal at 7 K/μL. The urinalysis was positive only for trace ketones and few bacteria. A point-of-care ultrasound of the right lower quadrant was performed by the emergency physician during which the appendix was not visualized. A small amount of intraperitoneal free fluid was identified, and a portion of small bowel was identified with an ‘onion skin’ (alternating hypo and hyperehoic) target appearance measuring 2.1 cm, suspicious for intussusception. The patient's pain and tenderness persisted, and after viewing the point-of-care ultrasound, the surgical team still requested a computerized tomography (CT) to be obtained. The CT of the abdomen and pelvis with oral and intravenous contrast was performed, revealing a normal appendix and small amount of free fluid along with a short segment of transient ileo-ileal small bowel intussusception. There was no bowel wall thickening or bowel obstruction. Contrast material was seen distally within the colon.

While in the ED, the patient received a fluid bolus of normal saline and morphine intravenously for analgesia. Initially, a surgical consult was called with concern for appendicitis. After the CT results were reviewed, the patient was reassessed. His abdomen was now non-tender to palpation. The patient tolerated fluids orally, and his condition continued to improve. Serial examinations continued for a total ED course of 7 h. There was no return of abdominal pain or tenderness. The patient was discharged home after arranging close follow-up with his pediatrician and discussion of return instructions. The final diagnosis of transient intussusception was made.

### Imaging

**Figure 1:**
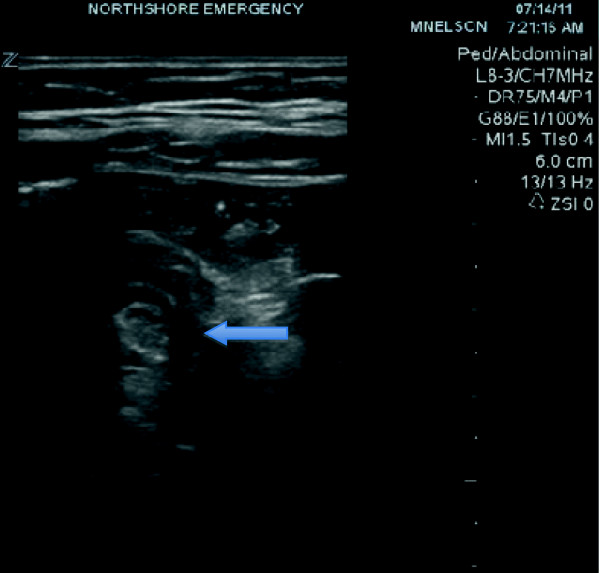
First US image of the right lower quadrant.

**Figure 2:**
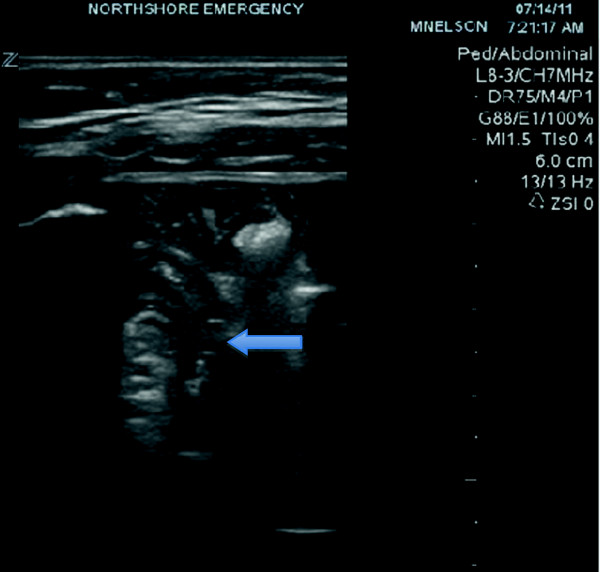
Second US image of the right lower quadrant.

Figures 1 and 2 and Additional file 1 present the image results of the bedside ultrasound (US).

### Discussion

Intussusception is a common gastrointestinal emergency in pediatric patients. It involves the invagination of a segment of small bowel into a segment of adjoined intestinal lumen and is the most common cause of small bowel obstruction in children. The incidence of intussusception is approximately 0.2% [2]. Male-to-female ratio is 3:1, increasing to 8:1 after the age of 4 years [3]. Two thirds of children with intussusception are less than 1 year old, most commonly affecting infants 5 to 10 months of age. Intussusception can occur in adults but is rare, accounting for 16% of the cases [3]. Frequently, the terminal ileum telescopes into the colon but colo-colic and ileo-ileal variants can also occur. Idiopathic intussusception typically occurs at the ileo-colic junction and often affects infants and toddlers. Enteroenteral intussusception often occurs in older children. Intussusception is affiliated with Henoch-Schonlein purpura, cystic fibrosis, hematologic dyscrasias, postoperative changes, or lead points.

Lead points are found in 2% to 12% of children, as age increases, so does the occurrence of a lead point, and the chance of a non-surgical reduction becomes less common [3]. Examples of lead points are Meckel's diverticulum, enlarged mesenteric lymph nodes, benign or malignant tumors, mesenteric cysts, and submucosal hematomas associated with HSP. Other possible causes are viral inducers including rotavirus, rotavirus vaccine, and common upper respiratory illnesses [4].

Small bowel intussusception occurs less frequently than other types. Classic presentation involves colicky abdominal pain, vomiting, palpable mass, and blood per rectum or currant jelly stools (occurring in 20% of patients) [5]. Early in the process, lymphatic drainage is impaired, and then, increased hydrostatic pressure within the wall of the intussusceptum decreases the venous return. If the intussusception and obstruction reaches high pressures, arterial flow also can be inhibited, causing necrosis. Ischemic mucosa sloughs off, causing heme-positive currant jelly stools. Transient small bowel intussusceptions can present with non-specific symptoms such as fever, abdominal pain, and irritability [6]. However, there are no reliable clinical signs and symptoms that can make the diagnosis. Small bowel intussusception does not frequently have the classic presentation seen with ileo-colic intussusception.

More than 50% of adult intussusceptions are small bowel intussusceptions that are commonly caused by malignancies [7]. In the pediatric population, however, transient small bowel intussusceptions are less common, and their signs and symptoms are not well described. Most pediatric small bowel intussusceptions resolve spontaneously and only require observation. Catalano described transient small bowel intussusception as momentary dysrhythmic contractions resulting in a functional abnormality of peristalsis. In the absence of an organic cause, a short segment of the small bowel does not contract normally, causing the balance of peristaltic forces to rotate the intestinal wall inward, creating the intussusceptum [8]. In a small prospective study of 108 real-time cases, 41 intussusceptions were identified on ultrasound and analysis of bowel wall motion indicated early resolution. These cases were followed with repeat ultrasonography at 30 min, 3 days, and then again at 2 weeks [9].

In general, transient small bowel intussusception occurs in older children with a mean age of 4 years. Certain factors are thought to predispose children to small bowel intussusception including bowel wall swelling, abnormal motility, and adhesions. Transient small bowel intussusception is more difficult to identify on ultrasound compared to ileo-colic intussusception because of atypical location and small diameter. The outer rim is thinner, overall diameter is smaller, there is less mesenteric fat, and no lymph nodes are often involved [10].

In general, it can be difficult to differentiate the type of intussusception on US. In a retrospective review of 49 cases over a 2-year time frame, Park et al. found that there were a few reliable criteria to distinguish between transient small bowel intussusception (TSBI) and the more common ileo-colic intussusception (ICI). First and foremost, ICI is more commonly (90%) located in the RUQ or epigastric region of the abdomen; whereas TSBI is (91%) located in the RLQ or periumbilical region [5]. Also, as stated earlier, the AP diameter and the outer rim of the intussusception in TSBI are 50% smaller than that found in ICI [5,10].

Ultrasound is highly accurate for the diagnosis of ileo-colic intussusception with a reported sensitivity of 98% to 100%; however, the diagnosis of small bowel intussusception is more difficult with an ultrasound detection rate approaching only as high as 84%. [5,6]. Ultrasound may illustrate small bowel intussusception as a crescent-in-doughnut or multilayered ‘onion skin’ round mass on a transverse scan and as the short segment sandwich sign on longitudinal scan [10]. In a prospective study involving 550 children with abdominal pain over 2 years, 21 of patients were found with SBIs in the right lower quadrant [11]. Another study of 49 cases of intussusception, 22 of which were small bowel and 27 were ileocecal diagnosed using ultrasonography. An intussusception length of >3.5 cm has been reported as a sensitive and specific predictor for surgical intervention, as compared to those that will resolve spontaneously [5,9].

CT scan findings of transient SBI have been described on rare occasion. However, intussusception with obstruction is well described. On CT, there are three findings that are characteristic: intraluminal soft tissue mass with an eccentric fat density due to invaginated mesentery (target pattern), reniform or bilobed mass with peripheral high attenuation due to thickened bowel wall (reniform pattern), and a sausage-shaped mass with alternating areas of high and low attenuation due to bowel wall, mesentery, and intestinal gas, fluid, or oral contrast (sausage-shaped pattern) [8,12]. CT scans can distinguish intussusception secondary to a lead point versus no lead point, therefore decreasing the incidence of unnecessary surgery [7].

## Conclusions

While CT scan provides superior anatomical detail, ultrasound remains as the primary imaging modality to diagnose and evaluate abdominal pain in children. Ultrasound is preferred because it is fast, non-invasive, and eliminates the growing concern of cumulative radiation. In children presenting to the emergency room with right lower quadrant pain, ultrasound is the preferred initial modality. Transient small bowel intussusception is an entity that may be encountered as more clinicians use point-of-care ultrasound to investigate abdominal pain. However, unless transient small bowel intussusception can be definitely identified as the source of the pain, care must be taken to avoid missing more pressing diagnoses such as appendicitis or ileo-colic intussusception.

## Consent

Informed consent was obtained from the patient's parent for publication of this report and any accompanying images.

## Competing interests

The authors declare that they have no competing interests.

## Authors' contributions

MN and TP were the treating physicians and performed the bedside ultrasound. MN, TP, and CR were all responsible for writing the case report. All authors read and approved the final manuscript.

## Supplementary Material

Additional file 1 US video of the right lower quadrant. Click here for file
